# A Scoring System for the Assessment of Quality of Care in the Management of Transfusion Dependent Thalassemia

**DOI:** 10.3390/hematolrep18040046

**Published:** 2026-07-01

**Authors:** Michael Angastiniotis, Lily Cannon, Androulla Eleftheriou

**Affiliations:** Thalassaemia International Federation, Ifigenias Street, 2007 Nicosia, Cyprusandroullaeleftheriou@cytanet.com.cy (A.E.)

**Keywords:** thalassaemia, quality of care, low resource settings

## Abstract

**Objective**: To identify criteria which can be used locally to assess the quality of care for thalassaemia patients, leading to quality improvement measures. In low-resource settings, there is often minimal support for services, and the investigations used in patient monitoring are very basic. In order to select standards which can serve quality assessment, consideration is given to what is available in most centres. Importance is given to the need for the local service provider to self-assess the quality of care according to evidence-based minimal standards. **Methods**: A search in the recent literature was performed to identify measures of quality care in thalassaemia, selecting those which can be used in resource-poor settings. They are then compared to the standards listed in internationally accepted guidelines. **Results:** Twelve criteria were selected based on the routine information recorded by most centres. These include the following: clinical criteria: mean age (excluding paediatric clinics), pre-transfusion Hb < 9 g/dL, serum ferritin, MRI availability, heart iron (where available) >20 ms, LIC (where available) <3 mg/kg dw, LIC > 15 mg/kg dw, combination chelation within the last year, and BMI < 18.5 kg/m^2^. Social criteria (for adults): completed tertiary education, married/cohabiting, and employed full or part-time. Each is assigned a score with a total range from 0 to 10. **Conclusions**: Annual scoring according to achievements allows service providers to compare with previous years and conclude which of the basic services need to be further upgraded to achieve quality improvement. Scoring also allows for comparison with standards published in international guidelines. The clear aim is to aim for higher scores each year, indicating better patient outcomes.

## 1. Introduction

Thalassaemia is a term referring to hereditary clinical syndromes affecting the haemoglobin molecule and resulting in severe anaemia. The globin chains of the molecule, mainly alpha and beta chains, are reduced or not produced, creating a deficit in one chain type and so an imbalance in the molecule, which leaves globin chains unpaired; precipitation of these free molecules in the red cell precursors in the haemopoietic tissues causes cell death and ineffective erythropoiesis. If severe, the patient becomes dependent on regular blood transfusions, which over time lead to iron overload mainly from the apoptosis of the transfused red cells, as well as the effects of ineffective erythropoiesis leading to destruction of red cell precursors.

Survival and quality of life can be excellent if the complex treatment modalities are available, accessible and affordable. Treatment is lifelong, and components of clinical management should meet standards according to internationally accepted guidelines [1,2,3]. However, these standards are not always met, even in high-resource settings [4]. The main treatment modalities are as follows:

1. Blood transfusions must be adequate in order to correct the anaemia and suppress ineffective erythropoiesis. This is achieved by maintaining pre-transfusion haemoglobin (Hb) at least above 9 mg/dL to promote growth and vitality, as well as survival [1,2,5]. Donated blood must also be compatible with the recipient and safe from viruses and other contaminants, minimising adverse reactions. This requires a transfusion service that relies on voluntary non-remunerated donations and is well-equipped to perform all the necessary tests to prevent adverse reactions and transmission of infections.
2. Iron overload is subsequent to increased absorption from food and from breakdown of transfused red cells; excess iron damages vital organs. One measure of iron load is serum ferritin, which should be kept under 1000 ng/mL [6]. The body does not have a mechanism to remove excess iron, and so iron chelation (removal of iron pharmaceutically) must be adequate to prevent iron toxicity to vital organs, while being alert to possible chelator toxicity. Daily treatment is a necessary prerequisite for effectiveness.
3. Monitoring of iron overload through various methodologies and for the early detection of organ involvement is essential so that adjustment of treatment can be timely. This is done by blood tests (serum ferritin) and by radiological methods, including MRI [7,8].
4. Multidisciplinary collaborations are needed to ensure protection and expert treatment of organ damage. Heart, liver and endocrine toxicity, in which all endocrine glands can be involved, but most commonly those involved in growth and sexual development, lead to hypogonadism, as well as hypothyroidism and diabetes. These complications are inevitable without timely detection and treatment.
5. Psychosocial support is offered at all ages, both by the treating team of doctors and nurses, and by professional psychologists and social workers. Social achievements such as education, personal relationships, employment and social integration are all contributors to a satisfactory quality of life, a reason for pursuing survival despite the difficult life routine.
6. Expert centres are a requirement for good outcomes, which can offer all the services briefly described above and follow a significant number of patients long-term so that clinical experience is accumulated. Such reference centres form a collaborating network with smaller peripheral centres. This can ensure equity in the management of the disease, reaching patients [9,10].
7. The availability of innovative and curative treatments. These include medications which can improve the clinical course, but also those which are expected to render patients transfusion independent. These therapies currently benefit only a small minority of patients because of unaffordability, but also because the process requires specialised infrastructure, making access difficult for most patients [11,12].

Even without the benefit of innovative therapies, well-treated patients adhering to conventional treatment now survive to old age, while having a productive family and working life [13,14]. In real life, patients fall into the following categories:

1. Those who are not treated at all. If they suffer from the severe transfusion-dependent form of thalassaemia, they will die in childhood, while those with less severe syndromes may survive for some years but with a poor quality of life.
2. Those who benefit from some basic blood transfusion usually maintain low pre-transfusion haemoglobin and have indications of iron overload and organ involvement despite the availability of iron chelation. Multidisciplinary care to monitor and offer timely intervention to organ damage is not easily found, and full health coverage is not available; such patients may survive to adolescence or young adulthood with increasing complications to vital organs. Unfortunately, this group represents the majority of the global patient population [15,16,17].
3. Those benefiting from full comprehensive care available under universal health coverage. Such a service can achieve long survival so that even severely affected patients can live into their 60s and enjoy near-normal lives. Many are now in the medical and other professions [13,14].
4. Patients who are cured through haemopoietic stem cell transplantation (HSCT) or gene therapy.

Most patients globally belong to the first two treatment categories because they live in poor/low-resource settings. Low-resource settings generally refer to those in which thalassaemia is classified as low priority by policy makers and disease-specific services are usually underfunded. This means that patients do not benefit from the optimal care as supported by international standards to achieve the outcomes which are evident in well-resourced settings. In addition, medical expertise and other manpower concerns may also be lacking, and in some such countries, there is no national policy to cover patients’ treatment. Lack of medical expertise is also a factor in many settings.

Considering the complexity of services required by thalassaemia, affordability is a major issue; where universal health coverage is adequate, the quality of services can be expected to be high. For example, in many countries in Asia, the direct medical cost per thalassaemia patient per year ranges from 400 to 1000 USD (mean patient age 20 years) [18,19,20]; in contrast, in countries like Greece, Italy and the United Arab Emirates (UAE) it is around 30,000 USD [21,22,23] (mean patient age 45 years) [13,14]. Similarly, the Universal Health Coverage Index (UHC)/Service Coverage Index (SCI) are >80 in Greece and Italy, while in Asia, most countries vary between 54 and 74 (from WHO tables in https://data.who.int/indicators/i/3805B1E/9A706FD, accessed on 19 March 2026).

In order to achieve quality improvement, each treatment unit requires an assessment of its services, with a recognition of weaknesses, so that corrective measures may be promoted either directly by the administrative authorities or through advocacy. Criteria of quality or effectiveness are based on best practice standards as described in [1,2,3].

## 2. Methodology

A search of the recent literature was performed to identify measures of quality care in thalassaemia. The inclusion criterion was to identify measures which can be used in a poor/low resource setting and can be easily applied by an administrator or a clinician serving thalassaemia patients.

One measure that has been published as a prognostic of patient outcomes and theoretically can be used as a measure of quality is the Thalassemia International Prognostic Scoring System (TIPSS) [24]. The TIPSS score formula is described as 1.4 × heart disease + 0.9 × liver disease + 0.9 × diabetes + 0.9 × sepsis + 0.6 × alanine aminotransferase ≥ 42 IU/L + 0.6 × haemoglobin ≤ 9 g/dL + 0.4 × serum ferritin ≥ 1850 ng/mL. TIPSS score thresholds of greatest differentiation were assigned as <2.0 (low-risk), 2.0 to <5.0 (intermediate-risk), and ≥5.0 (high-risk) for premature death. This may be used for the purposes of assessing the outcomes of service. However, the objective is to simplify so that a poor/low-resource centre may use the most available patient data. For example, in the TIPSS, the criteria for liver disease may not be clear, and diabetes prevalence depends on the age range.

Another published scoring system is the Mahidol score [25]. This is a scoring system consisting of 6 clinical criteria: haemoglobin at steady state (>7.5 6–7.5 <6 g/dL), age at receiving first blood transfusion (>10 5–10 <5 years), requirement for blood transfusion (none/rare, occasional, regular), size of spleen (<3 3–10 >10 cms), size of spleen, and growth expressed in centiles of height and weight. This was a system designed for classifying disease severity in individual patients with thalassaemia intermedia.

These two systems were not adopted since the consideration of this study is that treatment centres in poor/low-resource settings need simple indicators based on the services that they can afford and on patient outcomes that they can easily extract from their records.

The following indicators were assessed as quality-of-care standards:

1. Indicators of treatment standards:
  a. Blood adequacy: pre-transfusion Hb;
  b. Blood safety: allo-immunisation rate;
  c. Iron chelation adequacy: serum ferritin and the proportion of patients with SF < 1000 ng/mL;
  d. Iron chelation effectiveness: serum ferritin and the proportion of patients with SF > 2500 ng/mL;
  e. Monitoring of iron deposition in vital organs: availability of MRI (not available, rarely used, annual measurements of iron in heart and liver). If available: proportion of patients with MRI T2* (an MRI relaxation parameter used for iron assessment) heart >20 ms, and Liver Iron Concentration (LIC) <3 mg/kg dw;
  f. Out-of-pocket expenses required by patients/families (Yes/No);
  g. Patient registry maintained: data of all patients attending the clinic, including those with transfusion-dependent thalassaemia (TDT), non-transfusion dependent thalassaemia (NTDT), HbH, and other diagnostic categories, are entered into an electronic disease-specific patient registry;
  h. Iron chelation availability: availability of all 3 chelators;
  i. Proportion taking combination chelation.
2. Outcome measures:
  a. Age distribution/mean age;
  b. Body Mass Index (BMI);
  c. Comorbidities (see Table 1);
  d. Proportion of tertiary education;
  e. Proportion >18 y employed;
  f. Proportion single/married;
  g. Quality of life measures, if used.

In choosing these measures, the consideration is that they should be objective, measurable indicators, avoiding any subjective assessments.

Another consideration is that assessing health in thalassaemia, there will be different patterns of disability or poor health according to age group, as well as the quality of care.

Taking into consideration these indicators and outcome measures, the following criteria have been selected to create a scoring system for the assessment of the quality of care for transfusion-dependent thalassaemia patients.

Criteria selected:

Criterion 1—Age:

Age can be expressed as mean age, age distribution or age range. It is an indicator of patient survival in settings where adult patients are being treated. In paediatric departments, other indicators of quality care are used (e.g., achievement of growth milestones and limited skeletal deformities).

Centres are divided into those which see only paediatric patients (aged usually 1–18 years), those that see only adults (18+ years) and some that accept all ages.

Criterion 2—Pre-transfusion Hb:

We have chosen the proportion of patients whose pre-transfusion haemoglobin (PTHb) is below 9 g/dL as an indicator of poor prognosis. This is a measurement that is done routinely, even in any poor/low resource setting. In selected centres of excellence, the proportion is low; published data from centres in the West indicate 0–9% at this level (Italy [5], Greece [26]); in contrast, other countries report a high proportion of patients being kept at this low level, ranging from 60% to 100% (Bangladesh [27], Iraq [28], Sri Lanka [29], Indonesia [30], Maldives [31], and Pakistan [32]).

PTHb mean, or average, may be an easier measure in some settings.

Criterion 3—Serum ferritin SF:

The proportion of patients who maintain a serum ferritin level below 1000 ng/mL can indicate whether chelation control is effective: good chelation is reported when the numbers reporting <1000 ng/mL were 0–20%, with examples from Thailand [15], Greece (8.5% of patients) [26], Italy [33], and Cyprus [34]. Moderate control with 18–37% having <1000 was reported in Malaysia [35], Nepal [36], and Palestine [37]. Around 80% of patients with high ferritins were reported in Pakistan [32,38].

The proportion of patients with SF > 2500 is less accurate because the measure is sensitive to other factors such as inflammation.

Criterion 4—MRI measurements of iron load:

MRI is not universally available, and it is often offered as an out-of-pocket expense—not accessible and not affordable to most patients/families. For these reasons, a centre may just report the proportion of patients who have had a measurement (either in the heart or liver) in the last year and follow progress, if any, over the years.

If T2* of the heart is available, then the proportion of patients who measure >20 ms (low risk for heart failure) may be a good measure. We have looked at reports from 7 countries; these indicate a high proportion of patients with >20 ms (70–90.5%). In one study from Pakistan, only 27% of patients achieve this level [39].

LIC is a reflection of total body iron and is more difficult to control by chelating agents. Normal or safe values are those which measure <3 mg/kg dw. The proportion of patients who maintain this level is an indicator of good chelation practices. Low proportions were reported in a study from Tunisia (5%) [40]; good results were reported in Italy [33], Greece [26] and Cyprus [34], where 45–65% of patients had normal readings. Conversely, LIC > 15 mg/kg dw was reported to be high in Malaysia (39.4% of patients) [41], while Iran (4.4%) [42], Greece, Cyprus and the UK [43] report the same in 3–16% of patients.

Criterion 5—The use of combined chelation regimes:

This is an indication of availability as well as of concern by the clinical team of the need to reduce or rescue from the consequences of iron toxicity. Low usage was reported by Nepal (1%) [36] and Iraq (0.7%) [28]. In these countries, the chelating agents are not in constant supply, and so there is interrupted use by patients.

Criterion 6—Body Mass Index (BMI):

This is an indicator of poor growth due to possible under-transfusion and possible malnutrition. Measures <18.5 kg/m are an indicator of poor growth and are reported by Thailand (30% of patients) [15], Saudi Arabia (41%) [44], and Vietnam (28%) [45]. In contrast, Greece reported only 1.5% of patients below this level [26].

Criterion 7—Education in adult patients (>18 years):

Achieving a tertiary education is an indicator of good clinical management, survival, and a good quality of life. Patients (26–55%) from the USA [46], Oman [47], UAE [48], Cyprus [34], and Lebanon [49] achieved this level. However, in Iran [50], Iraq and Indonesia [30], only 7–17% achieved this.

Criterion 8—Marriage:

An indicator of social integration and personal adjustment.

Criterion 9—Employment:

An indicator of social integration and acceptance.

These indicators and suggested scores are listed in Table A1.

## 3. Discussion

Most of the published literature focuses on criteria and application of standards of care which are suitable for academic and reference centres that manage thalassaemia patients [1,2,3]. Most patients in high-prevalence countries across the world are, in fact, managed in peripheral non-academic units. In the Indian subcontinent, many treatment centres are driven by charity non-governmental organisations (NGOs), which struggle to reach the standards and quality of care described in evidence-based guidelines. This inequality results in poor outcomes while many treating units and patient support groups battle to improve results through advocacy and other supportive measures. Other health priorities, such as malnutrition and infectious diseases, overshadow chronic hereditary disorders. Because of high prevalence, however, corrective measures within the healthcare systems should be introduced [51,52].

Quality of long-term care is the degree to which care services contribute to maximising well-being and quality of life and increase the likelihood of personal and health outcomes that are consistent with the individual preferences, human rights and dignity of both patients and their caregivers. Optimal care is that which ensures long life compared to inadequate treatment and no treatment. For a service to be effective, people-centred services including safety considerations, informed choice, timely treatment (including flexible transfusion sessions), equitable services (considering gender and ethnicity), and, importantly, integrated multidisciplinary care are required.

A tool for self-assessment for treatment centres is suggested here, based on measurements which are mostly routine. Some, such as those based on magnetic resonance imaging, modified to measure iron load in the heart and liver, are in fact not available to many services in settings where funds are limited. However, these are becoming essential tools to support good outcomes and should be pursued by all service providers. Small peripheral clinics may address this need by networking with the nearest reference centre.

This scoring is not meant to be an epidemiological tool or for comparison between countries. It is a simple self-assessment tool for each treatment centre to evaluate from year to year whether standards are being met and what remains to be improved. Voluntary comparison with other centres in the country, or with published national data, is possible with self-improvement always in mind. We have stayed clear of more complex measurements like DALYs, QALYs and disability weights, as used by the Global Burden of Disease project, which may be difficult for the individual centre to easily complete.

The number of deaths per year in each centre should, of course, be recorded along with causes of death, but such information cannot readily be included in such a scoring system.

To understand the quality of care, the physician in charge needs to be fully aware of other influences on quality, such as:

- The health system within which the service operates: is there adequate support from administration, is there understanding and prioritisation of chronic hereditary disorders, and are there other pressing priorities to the health system (such as malnutrition, infectious diseases)? One major concern is the provision of healthcare coverage for these chronic conditions, either as part of a universal coverage system for the whole population or through a specialised insurance for such conditions.
- The socio-economic environment, including the educational level of the people, the income level compared to the out-of-pocket expenses, and the complexity of the population structure (ethnic minority groups, religious groups, cultural diversity, and recent migrants). Such subgroups can make service provision more complex, with additional needs such as the need for translations, reaching tribal communities, and raising awareness. Education and culture have been shown to influence the acceptability of certain services, such as prevention policies, adherence to treatment, and utilisation of services.

With these factors in mind, the physician in charge of a service for thalassaemia must consider advocacy, targeting healthcare authorities for improved support for the service, along with raising community support through patient associations. At the same time, more consideration of psychosocial support for patients and families may be necessary.

Blood disorders such as these require complex services, which, if not offered to the optimal level, result in increasing complications and premature death. Offering such services requires support from the national competent authorities, the medical team and the community. The suggested scoring system is only a tool for the medical team, who must ensure continual improvement at the clinical level. Quality improvement requires much more.

## 4. Conclusions

It is recommended that recognition of deficits in service provision should be documented annually in order to assist quality improvements. In this study, we suggest a list of items to be recorded each year for this purpose. These are readily available from patient records. In some situations, medical personnel will require training to ensure the expertise required to achieve the outcomes that are aimed for.

## Figures and Tables

**Table 1:** Major comorbidities in thalassaemia patients by age.

Childhood (<12 Years)	Adolescence (13–17 Years)	Adulthood (>18 Years)
The effects of anaemia	Iron overload	All endocrinopathies
Growth failure	Delayed puberty	Cardiomyopathy
Skeletal deformities and fractures	Onset of other endocrinopathies	Bone pain/osteoporosis
Splenomegaly		Liver disease

## Data Availability

No new data were created or analyzed in this study. Data sharing is not applicable to this article.

## Abbreviations

The following abbreviations are used in this manuscript:

Hb	Haemoglobin
MRI	Magnetic Resonance Imaging
LIC	Liver Iron Concentration
BMI	Body Mass Index
HSCT	Haematopoietic Stem Cell Transplantation
NGOs	Non-Governmental Organisations
USD	United States dollars
UHC	Universal Health Coverage
SCI	Service Coverage Index
WHO	World Health Organization
TIPSS	Thalassaemia International Prognostic Scoring System
SF	Serum Ferritin
TDT	Transfusion-Dependent Thalassaemia
NTDT	Non-Transfusion Dependent Thalassaemia
HbH	Haemoglobin H
PTHb	Pre-Transfusion Haemoglobin
DALYs	Disability-Adjusted Life Years
QALYs	Quality-Adjusted Life Years
UAE	United Arab Emirates
USA	United States of America
UK	United Kingdom
IU/L	International Units per Litre
T2*	T2-star (MRI relaxation parameter used for iron assessment)
dw	dry weight
wt	weight

## Appendix A

**Table A1:** Scoring system.

Criterion	0 Points	5 Points	10 Points
Mean age (excluding paediatric clinics)	0–20 years	21–30 years	31+ years
Proportion of patients pre-trans Hb < 9 g/dL	50–100%	10–49%	0–9%
SF	>1500 ng/mL (50+% patients)	1000–1499	<1000 (0–20% of patients)
MRI availability	Not available	Available with a fee	
Available occasionally	Available for free use according to guidelines		
Heart iron (where available) >20 ms	<20% of patients	20–40% of patients	50% of patients
LIC (where available) <3 mg/kg dw	<20% of patients	20–40% of patients	50% of patients
LIC > 15 mg/kg dw	>50%	20–40%	<20% of patients
Combination chelation within the last year	0–10% of patients		>10% of patients
BMI < 18.5 kg/m^2^	>10% of patients		<10% of patients
Completed tertiary education	<10% of patients	10–30%	>30%
Married/cohabiting	<10% of patients	10–30%	>30%
